# Convenient chirality transfer from organics to titania: construction and optical properties†‡

**DOI:** 10.1039/c8ra02926a

**Published:** 2018-04-30

**Authors:** Xin-Ling Liu, Ken Murakami, Hiroyuki Matsukizono, Seiji Tsunega, Ren-Hua Jin

**Affiliations:** Department of Material and Life Chemistry, Kanagawa University 3-2-7 Rokkakubashi Yokohama 221-8686 Japan rhjin@kanagwa-u.ac.jp

## Abstract

Polyethyleneimine (PEI) complexed with chiral d- (or l-) tartaric acid (tart) in water can self-organize into chiral and crystalline PEI/tart assemblies. It has been previously confirmed that the complexes of PEI/tart could work as catalytic/chiral templates to induce the deposition of SiO_2_ nanofibres with optical activity but without outwards shape chirality such as helices. In this work, we found that the templating functions of PEI/tart were still effective to prompt the deposition of TiO_2_ to form chiral PEI/tart@TiO_2_ hybrid nanofibres under aqueous and room temperature conditions within two hours. Furthermore, the co-deposition of TiO_2_ and SiO_2_ was also fulfilled to yield chiral PEI/tart@TiO_2_/SiO_2_ nanofibres. These TiO_2_-containing hybrid nanofibres showed non-helical shapes on the length scale; however, chiroptical signals with mirror relation around the UV-Vis absorption band of TiO_2_ remarkably appeared on their circular dichroism (CD) spectra. By means of the protocols of XRD, TEM, SEM, UV-Vis, CD and XPS, structural features and thermoproperties of the chiral TiO_2_ and SiO_2_/TiO_2_ were investigated.

## Abstract 2

Polyethyleneimine (PEI) complexed with chiral d- (or l-) tartaric acid (tart) in water can self-organize into chiral and crystalline PEI/tart assemblies which can prompt titania deposition and impart their chirality to the resulting titania.

## Graphical abstract

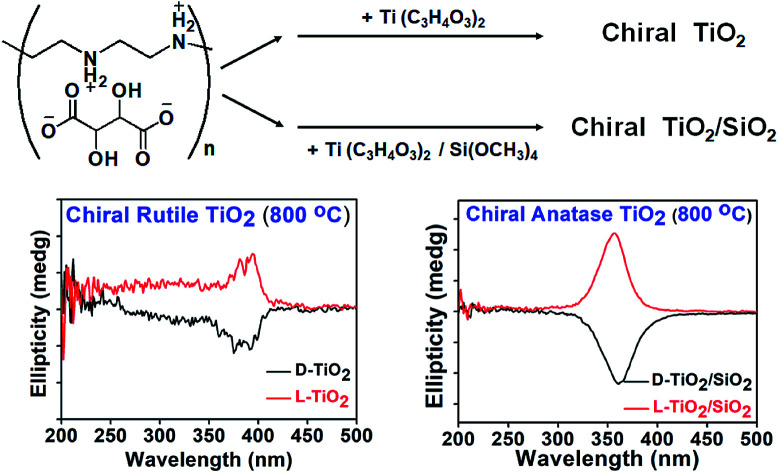

## Introduction

Recently, the synthesis of chiral inorganic nanomaterials has been a burgeoning topic in the research area of chirality.^1–3^ Chiral objects are non-superimposable with their mirror image, which can be caused by the asymmetric arrangement of their constituent units (atoms, molecules, nanoparticles, *etc.*) on different length scales. The combination between the chiral/asymmetric features and the diversely intrinsic properties of inorganics can result in novel catalytic, optical, electronic, and magnetic properties and applications such as in asymmetric catalysis, enantioselective separation, sensors, optical filters, enhanced surface-enhanced Raman scattering, and long-wavelength chiroptical activity.^4–12^ Therefore, it is interesting and challenging to synthesize chiral inorganic nanomaterials especially those crystalline materials with asymmetric space groups. To date, the synthesis and properties of chiral plasmonic metals (*e.g.*, Au, Ag), semiconducting quantum dots (*e.g.*, CdSe, ZnS) and metal oxides (*e.g.*, SiO_2_) have been widely researched. The interest has been continuously extended to other inorganic nanomaterials such as Si, ZrO_2_, ZnO, CuO, Y_2_O_3_, Ta_2_O_5_, Ln(OH)CO_3_ and Lu_2_Si_2_O_7_.^13–20^ Undoubtedly, these research studies will contribute to the development of the scope of chirality beyond the traditional organic chemistry.

Herein, we are especially interested in chiral titanium dioxide (TiO_2_). TiO_2_ is a kind of important semiconductor with a wide range of applications such as photocatalysts, pigments, cosmetics, solar cells and lithium ion batteries.^21–23^ Compared to chiral silica, there is not extensive attention for chiral properties of TiO_2_, only a few reports involved their preparation and potential applications. Generally, the synthetic chiral TiO_2_ could be divided into two types according to their synthesis procedures: the first one is the helix-shaped TiO_2_ with various helix pitches from tens nm to several μm; the other is the TiO_2_ with chiral cavities on the molecular scale prepared *via* molecular imprinting. For the helical TiO_2_, they are usually synthesized by the sol–gel process in the presence of helix-shaped templates, which included soft templates (*e.g.*, cholesterol gelator, lipid amphiphilic *N*-acylamino acids, *trans*-(1*R*,2*R*)- and *trans*-(1*S*,2*S*)-1,2-diaminocyclohexane derivatives, valine-derived chiral cationic gelators)^24–28^ and hard templates (*e.g.*, helical carbon nanotubes, SiO_2_ films with a long-range chiral nematic structures).^29,30^ Moreover, the glancing angle deposition (GLAD) (a kind of physical vapor deposition) has been also employed for the preparation of helical TiO_2_ films.^31,32^ For the molecularly imprinted TiO_2_, small chiral organic molecules (*e.g.*, l-phenylalanine, *R*-2-(4-isobutylphenyl)-propionic acid, (*S*) or (*R*)-2-phenylbutanoic acid) were mixed with the TiO_2_ sources during the formation of TiO_2_ and finally removed to produce chiral cavities by memorizing the configurations of chiral molecules.^33,34^ In addition, some potential applications of these chiral TiO_2_ have been demonstrated. For example, the surface plasmon resonance (SPR) of plasmatic Ag and Ag/AgCl would be enhanced when deposited on helical TiO_2_ and thus improved the visible-light photocatalytic performance of plasmatic-metal/TiO_2_ composites.^10^ Some chiral TiO_2_ films were able to detect circularly polarized light or enantioselectively recognize specific chiral small organic molecules.^32,33^

TiO_2_ possesses amorphous and crystalline (anatase and rutile) phases, of which amorphous TiO_2_ is generally formed at a low temperature and then transformed into crystalline anatase TiO_2_ by heating (usually < 600 °C) and further into rutile TiO_2_ with increasing the temperature (≥600 °C). Most previously reported chiral TiO_2_ were amorphous or anatase TiO_2_ obtained at the temperature less than 600 °C. However, the formation of chiral rutile or anatase TiO_2_ at a high temperature over 700 °C is still a challenge, which may be due to the following two reasons: (1) the phase-transformation temperature for the appearance of rutile TiO_2_ is high, at which the helical shapes or chiral cavities found on many chiral TiO_2_ may be destructed and thus the chirality could not be maintained; (2) anatase TiO_2_ is metastable under lower temperature but transformed into rutile TiO_2_ at a higher temperature. To overcome the first issue, it is expected to develop novel chiral TiO_2_ which possesses chirality not depending on the helical outward or metastable chiral cavities. For the second issue, anatase TiO_2_ can be stabilized by some special strategies such as decreasing the sizes, as the nano-sized anatase TiO_2_ may show improved thermal stability than that of the larger-sized one.^35–37^

Although the definition of chirality is quite simple, the expression of chirality varies with sizes, phases and shapes. This offers various possibilities to develop new inorganics with different chiral features to satisfy different demands.^15,38^ Herein, to address the issues on chiral TiO_2_ mentioned above, we developed a new way to prepare chiral TiO_2_ and TiO_2_/SiO_2_ composite nanofibres without helical shapes by using chiral templates self-assembled from polyamine and chiral tartaric acid. In our earlier work, it was confirmed that polyethylenimine (PEI) could complex with chiral d- (or l-) tartaric acid (tart) to form crystalline PEI/tart complexes with optical activity, and could be used as catalytic chiral templates to prepare non-helical SiO_2_ nanofibres with high-temperature-resistant (up to 900 °C) chirality.^39^ In the present work, we found that mixing PEI/tart complexes with TiO_2_ sources (titanium bislactates) at room temperature for 2 hours could easily result in deposition of TiO_2_ on the PEI/tart to form hybrids PEI/tart@TiO_2_. Also, when TiO_2_ and SiO_2_ sources were simultaneously used, co-deposition of TiO_2_/SiO_2_ occurred on PEI/tart to form hybrids of PEI/Tart@TiO_2_/SiO_2_. Both the hybridized nanofibres showed non-helical shapes but exhibited chiroptical signals on their circular dichroism (CD) spectra corresponding to the absorption bands of TiO_2_. After calcinations of PEI/tart@TiO_2_ at 500–800 °C, the phase of TiO_2_ was transformed into anatase TiO_2_ (500 °C) and finally into rutile TiO_2_ (800 °C), accompanied with the morphological change from nanofibres to nanoparticles (NPs). However, CD optical activity was still found on these calcined samples including the rutile TiO_2_ NPs. In contrast, in the case of PEI/tart@TiO_2_/SiO_2_, the nanofibrous morphologies were much less influenced by the calcination temperature, and sub-10 nm anatase TiO_2_ NPs were homogeneously distributed on the nanofibres even calcined at 800 °C. To the best of our knowledge, it is first example that chiral TiO_2_/SiO_2_ composites were successfully prepared by a one-step way and chiral anatase TiO_2_ were well maintained at such a high temperature of 800 °C.

## Results

**Scheme 1:**
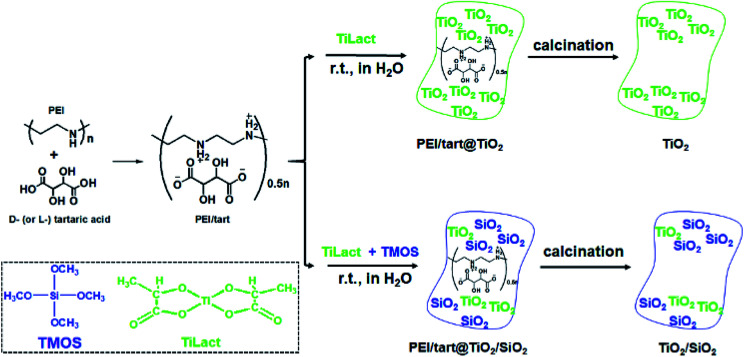
Synthetic procedures for chiral TiO_2_- (top) and TiO_2_/SiO_2_-related products (bottom) by the hydrolysis and condensation of titanium bislactate (TiLact) and tetramethoxy silane (TMOS) in the presence of PEI/tart complexes resulted from polyethyleneimine (PEI) and tartaric acid (tart).

It has been found that linear polyethyleneimine (PEI, (–NHCH_2_CH_2_–)_*n*_) can crystallize in water and thus form a series of assemblies with controllable nano/micro-morphologies^2,39^ and the amine groups on PEI can effectively catalyse the hydrolysis and condensation of alkoxy silane to induce the site-selective deposition of SiO_2_ around the surface of the PEI assemblies. Furthermore, PEI could complex with chiral tartaric acid (tart) to form crystalline polymeric complexes of PEI/tart with chiroptical activity, which still functioned as catalytic template for SiO_2_ deposition to generate chirality in the resulting silica frame.^40^ Besides SiO_2_, one-dimensionally (1-D) nanostructured PEI@TiO_2_ powders and films could be prepared by the accelerated hydrolysis–condensation of titanium bislactates (TiLact) in the presence of crystalline PEI assemblies.^41,42^ Herein, we are interested to probe whether PEI/tart could be also applicable as chiral template in preparation of chiral TiO_2_ and TiO_2_/SiO_2_ nanomaterials. For this purpose, two synthetic procedures were designed and shown in Scheme 1. In the first one, only the TiO_2_ source of titanium bislactate (TiLact) was mixed with PEI/tart to produce PEI/tart@TiO_2_ hybrids; in the second one, both TiO_2_ source (TiLact) and SiO_2_ source (tetramethoxy silane, TMOS) were simultaneously used to co-deposit TiO_2_/SiO_2_ on the templates of PEI/tart. Finally, these hybrids were treated by calcination at different temperatures (500–800 °C) to produce TiO_2_ and TiO_2_/SiO_2_, respectively.

### Characterizations of PEI/tart@TiO_2_ and TiO_2_

**Fig. 1:**
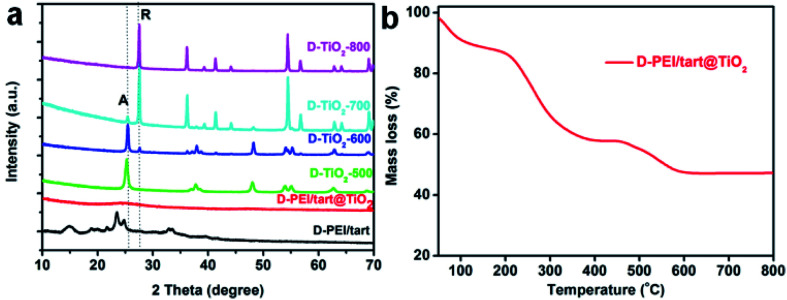
(a) XRD patterns for d-PEI/tart, d-PEI/tart@TiO_2_ (before calcination), and d-TiO_2_-*X* (after calcination, calcination temperature *X* = 500, 600, 700 and 800 °C); (b) TG curve for d-PEI/tart@TiO_2_.

As typical examples, the XRD patterns of d-form samples including template, as-prepared hybrid and calcined forms were shown in Fig. 1a. The complexes of d-PEI/tart exhibited several diffraction peaks in the 2*θ* range of 10–40 degree demonstrating the crystalline feature of PEI/tart complexes.^40^ Whereas, only a broad halo peak between 20–30 degree was found on d-PEI/tart@TiO_2_. After calcination at a high temperature, these hybrids turned into crystalline TiO_2_ products. Two kinds of phases for TiO_2_ were detected, including the anatase TiO_2_ (JCPDS card no. 21-1272) with the characteristic peak (marked with A) around 25.3 degree and the rutile TiO_2_ (JCPDS card no. 21-1276) with the peak (marked with R) around 27.5 degree. The phases changed with the temperature: only anatase TiO_2_ appearing at 500 °C, both anatase and rutile TiO_2_ at 600 and 700 °C, and only rutile TiO_2_ at 800 °C. Meanwhile, the peak A for anatase TiO_2_ decreased from 500 to 700 °C and finally disappeared at 800 °C while the peak R for rutile TiO_2_ appeared at 600 °C and further increased with temperature. These changes of peak A and R showed the phase transformation from anatase to rutile, which is a common phenomenon found on many crystalline TiO_2_ products during heating. That is the rutile is the thermodynamically stable phase while the anatase is a metastable one. According to the TG curves (Fig. 1b), the mass ratio of organic components in d-PEI/tart@TiO_2_ was about 41% based on the mass loss between 150 and 800 °C. The XRD patterns and TG curves for the l form products are close to those of the corresponding d-form products and thus were not shown here. The XRD and TG results preliminarily demonstrated that the PEI/tart complexes could induce the deposition of TiO_2_.

**Fig. 2:**
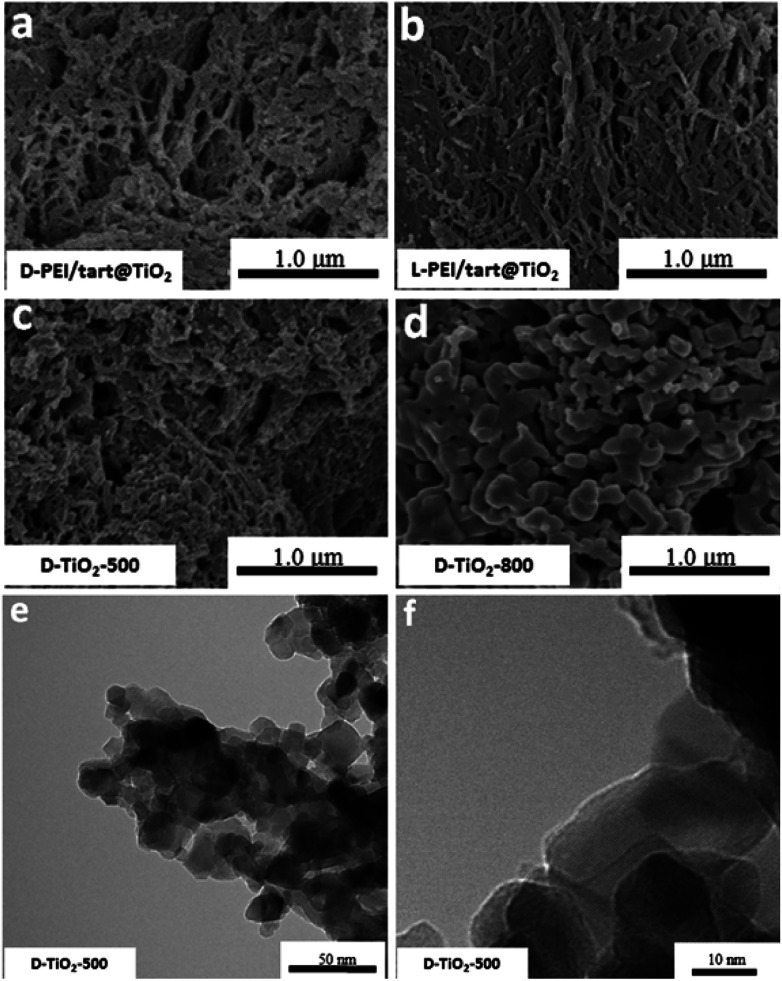
SEM image for (a) d-PEI/tart@TiO_2_, (b) l-PEI/tart@TiO_2_, (c) d-TiO_2_-500 and (d) d-TiO_2_-800; (e and f) the TEM images for d-TiO_2_-500.

The sizes and nano/microscale morphologies of these TiO_2_-related samples were visualized by SEM and TEM (see Fig. 2). For d-PEI/tart@TiO_2_ (Fig. 2a) and l-PEI/tart@TiO_2_ (Fig. 2b), nanofibres bundles (average diameter ∼50 nm, length *ca.* 5 μm) were observed. Thus, the crystalline PEI/tart assemblies were still effective to produce 1-D TiO_2_ nanofibres under the aqueous and mild conditions. Similar to our early case in chiral silica deposition, there are also no shape chirality (*i.e.*, helical shape) for the d- and l-PEI/tart@TiO_2_. After calcinations, the morphologies for d- and l-form samples were still similar to each other, and hence only the d-TiO_2_ sample's images in SEM and TEM were displayed in Fig. 2c–f. The nanofibres observed from as-prepared powders of d-PEI/tart@TiO_2_ (Fig. 2a) were destroyed when they were calcined at 500 °C (d-TiO_2_-500, Fig. 2c), and finally turned into irregular nanoparticles (NPs) with sizes of about 100–300 nm after treated at 800 °C (d-TiO_2_-800, Fig. 2d). This morphological change was also supported by the TEM images of d-TiO_2_-500 (Fig. 2e and f), where NPs with crystal lattice fringes and sizes about 10–25 nm were observed.

### Characterizations of PEI/tart@TiO_2_/SiO_2_ and TiO_2_/SiO_2_

**Fig. 3:**
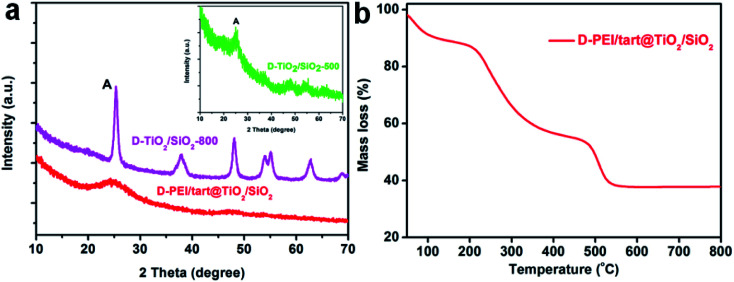
(a) XRD patterns for d-PEI/tart@TiO_2_/SiO_2_ (red line), d-TiO_2_/SiO_2_-500 (green line, inset) and d-TiO_2_/SiO_2_-800 (magenta line); (b) the TG curve for d-PEI/tart@ TiO_2_/SiO_2_.

The samples obtained from the process of co-deposition of silica and titania were also characterized by the same methods. The XRD patterns for TiO_2_/SiO_2_-related samples were shown in Fig. 3a. Similar to d-PEI/tart@TiO_2_, the as-formed d-PEI/tart@TiO_2_/SiO_2_ hybrids were still amorphous. Compared with d-TiO_2_-500 (calcinated at 500 °C), the intensities of peaks for the anatase phase on d-TiO_2_/SiO_2_-500 were very weaker (Fig. 3a, inset). After heating at 800 °C, the intensities were obviously increased. However, different from the phase of rutile TiO_2_ found on d-TiO_2_-800, only anatase TiO_2_ was identified on d-TiO_2_/SiO_2_-800. The mass ratio of organic component in d-PEI/tart@TiO_2_/SiO_2_ estimated by the TG curve was about 51% (Fig. 1b).

**Fig. 4:**
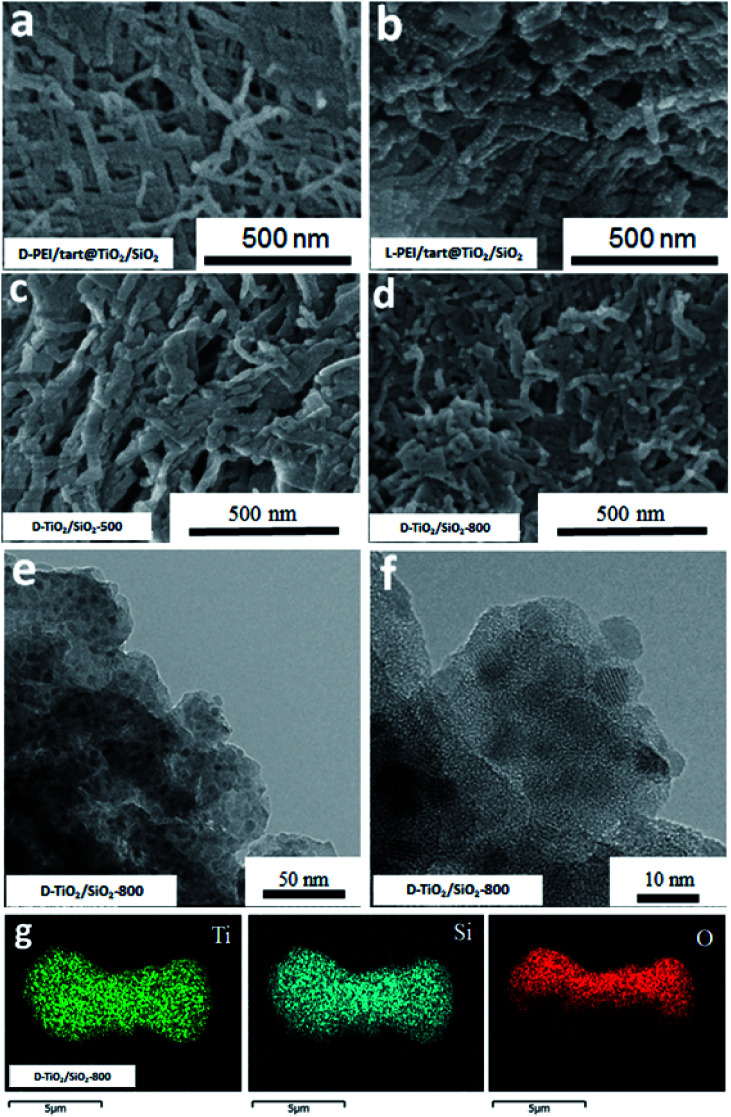
SEM image for (a) d-PEI/tart@TiO_2_/SiO_2_, (b) l-PEI/tart@TiO_2_/SiO_2_, (c) d-TiO_2_/SiO_2_-500 and (d) d-TiO_2_/SiO_2_-800; (e and f) the TEM images for d-TiO_2_/SiO_2_-800; (g) the elemental mapping of d-TiO_2_/SiO_2_-800 (green for Ti, blue for Si, red for O).

Furthermore, from the SEM images, the nanofibres were observed on the d- and l-PEI/tart@TiO_2_/SiO_2_ (Fig. 4a and b), similar to those for d- and l-PEI/tart@TiO_2_ hybrids (Fig. 2a and b). Different from the d-TiO_2_ samples, the nanofibres were effectively maintained on d-TiO_2_/SiO_2_-500 (Fig. 4c) and even d-TiO_2_/SiO_2_-800 (Fig. 4d) which were sintered at heating condition. From the TEM images of TiO_2_/SiO_2_-800 (Fig. 4e), many nanoparticles (NPs) less than 10 nm were observed; on the high-magnification TEM image (Fig. 4f), lattice fringes were clearly observed on these sub-10 nm NPs. It is clear that these TiO_2_ NPs were surrounded by the amorphous SiO_2_. Moreover, the elemental mapping (Fig. 4g) showed that Ti, Si and O were homogeneously mixed. These results implied that the co-deposition of TiO_2_ and SiO_2_ proceeds effectively on PEI/tart and the resulting structures of the encapsulated TiO_2_ in silica frames prevent the phase transformation of the component of TiO_2_ from anatase to rutile with maintaining the 1-D nanofibrous morphology even at 800 °C calcination.

### CD optical activity of TiO_2_ and TiO_2_/SiO_2_-related products

**Fig. 5:**
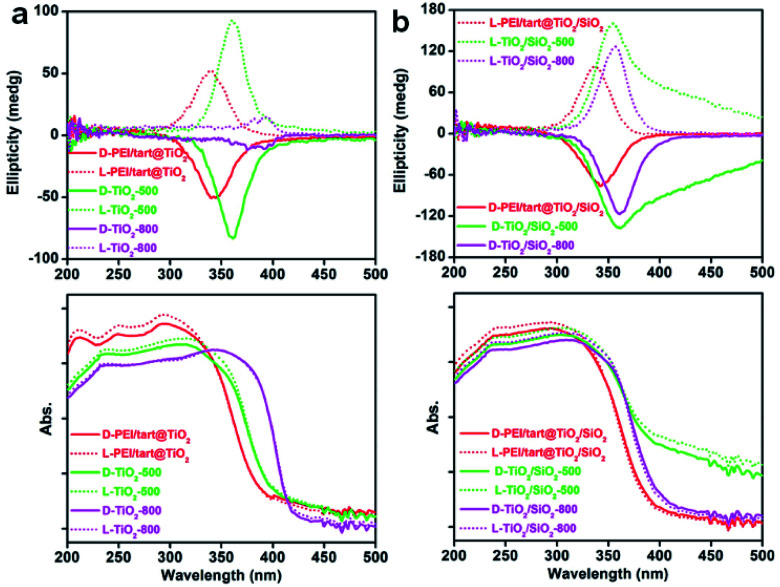
The DRCD (top) and UV-Vis absorption (bottom) spectra for (a) TiO_2_-related samples and (b) TiO_2_/SiO_2_-related samples (solid lines for d-form samples and dotted lines for l-form ones; red lines for the hybrids; green and magenta lines for the samples calcined at 500 and 800 °C, respectively).

The powders of TiO_2_ and TiO_2_/SIO_2_ were conducted to CD spectroscopy. Fig. 5 showed the solid-state diffuse reflectance circular dichroism (DRCD) and corresponding UV-Vis absorption spectra of PEI/tart@TiO_2_. Although no any helical shape images were observed on the nanofibres-like morphologies (SEM images, Fig. 2a and b) for the d- and l-PEI/tart@TiO_2_, a pair of mirror relationship lines appeared clearly on the CD spectra with the antipodal signals across 280–420 nm and centred around 340 nm: the CD signal is negative for d-PEI/tart@TiO_2_ while positive for l-PEI/tart@TiO_2_ (Fig. 5a). The d- and l-PEI/tart@TiO_2_ showed similar UV-Vis absorption spectra with strong absorption from 200 nm to 400 nm, which is attributed to the electronic transition from the valence band to the conduction band of TiO_2_. With increasing the calcination temperature, the peaks for the CD signals showed a red-shift to 360 nm (at 500 °C) and then to 392 nm (at 800 °C), accompanied with the red-shift of the UV-Vis absorption extending to 420 nm. Generally, the band gap of TiO_2_ decreases from amorphous to anatase to rutile TiO_2_, and correspondingly the UV-Vis absorption band shifted to the long wavelength (red shift).^21^ According to the phase-changes seen in the XRD patterns (Fig. 1a), the red shift on the CD signals and UV-Vis absorption would be attributed to the phase transformation from amorphous to rutile. Such phase transformation also influenced the intensities of CD signals of the chiral products as order of TiO_2_-800 (rutile) < PEI/tart@TiO_2_ (amorphous) < TiO_2_-500 (anatase).

For the case of co-deposited system, DRCD spectra (with signals centering at 340 nm) and UV-Vis absorption spectra (200–400 nm) of PEI/tart@TiO_2_/SiO_2_ still resemble those of PEI/tart@TiO_2_ (Fig. 5b). Since silica itself has no characteristic absorption band between 200 and 800 nm, the as-observed CD signals were dominated by the TiO_2_ components. After calcination the hybrid form at 500 °C, red-shift was observed both on the CD signals (to 360 nm) and UV-Vis absorption (to 420 nm) of TiO_2_/SiO_2_-500, which is similar to those of TiO_2_-500. The tailing lines towards longer wavelength over 400 nm would be due to the presence of organic byproducts stuck in the silica frame which formed during 500 °C calcination. Because no phase transformation (from anatase to rutile) occurred after calcination at 800 °C, the CD and UV-Vis spectra of TiO_2_/SiO_2_-800 are similar to those of TiO_2_/SiO_2_-500 with disappearance of the tailing lines. In addition, the CD intensity increased in the order of PEI/tart@TiO_2_/SiO_2_ < TiO_2_/SiO_2_-800 < TiO_2_/SiO_2_-500.

## Discussion

Our previous research has proved that PEI/tart complexes could give nanofibrous PEI/tart@SiO_2_ hybrids and their derived SiO_2_ nanofibres after calcinations at 500 °C or above. The present results confirmed that the PEI/tart complexes are still effective to produce PEI/tart@TiO_2_ hybrids nanofibres. However, after calcinations at 500–800 °C, the nanofibrous morphologies of PEI/tart@TiO_2_ were destroyed. Former silica is amorphous even after calcination but the later titania becomes to crystalline structure from the amorphous state of the as-prepared form when heated. Therefore, there would arise strong strain which induces re-structuring the morphology of the hybrids. Moreover, after titania deposition, the crystalline template PEI/Tart lost its initial crystallinity although fibrous morphology was directed (Fig. 1a). This is different to the case of silica deposition in which the template component PEI/Tart is encapsulated in the produced fibrous hybrid of PEI/Tart@SiO_2_ and remains its crystalline structure some degree.^40^ This difference relates to the different feature of carboxylate on silica and titania. Usually, the carboxylate can interact with metal ions of oxide with coordination interactions while such interaction does not occur with silica.^46^ Probably, the metastable nanofibrous hybrid PEI/tart@TiO_2_ is a loose accumulation of the components of PEI, Tart and amorphous TiO_2_ so that at high temperature the fibrous morphology was destroyed with accompanying the growth of crystallites of TiO_2_.

Although various chiral templates have been developed for the synthesis of chiral SiO_2_, the use of these templates in the synthesis of chiral TiO_2_ and TiO_2_/SiO_2_ composites are still limited. There was an example of a two-step method to prepare chiral TiO_2_/SiO_2_ films: SiO_2_ film with a long-range chiral nematic structure was firstly synthesized, then immersed into TiCl_4_ solution and finally calcinated at 600 °C to form chiral SiO_2_/anatase TiO_2_ film.^29^ In the present work, since both SiO_2_ and TiO_2_ could be deposited on the fibrous template of PEI/tart, it offered the feasibility for the co-deposition of TiO_2_ and SiO_2_ by an alternative one-step way. As confirmed by the elemental mapping in Fig. 4g, this method afforded homogeneous distribution between the as-formed TiO_2_ and SiO_2_ during the formation process, which resulted in better morphological stability of TiO_2_/SiO_2_ than that of the sole TiO_2_ system since the SiO_2_ nanofibres were thermally stable. As shown in the TEM images in Fig. 4e and f, it is clear that on the TiO_2_/SiO_2_ nanofibres, sub-10 nm TiO_2_ NPs were distributed on silica matrices. Consequently, the diffusion, aggregation and rearrangement of Ti and O atoms during calcinations would be restricted by the surrounding linkage of Si–O–Si–O, which are strong barriers to growth and phase transformation of TiO_2_ NPs. Indeed, even after heating at 500 °C, the crystallinity of TiO_2_ was still weak as judged by the XRD pattern (Fig. 3a), and a higher temperature (800 °C) was needed to facilitate the diffusion and rearrangement of Ti and O atoms to form anatase TiO_2_ with improved crystallinity. Although rutile TiO_2_ is a thermodynamically stable phase in the bulk state, some theoretical and experimental evidences have confirmed that anatase TiO_2_ is in fact stable when the sizes are small (less than 14 nm).^35–37^ Hence, both the anatase phase and the CD optical activity of TiO_2_ NPs were maintained even at 800 °C on TiO_2_/SiO_2_.

CD optical activity was found on all these TiO_2_-related products in spite of the morphological and phase change, from which it could be inferred that the chirality of TiO_2_-based products was much less dependent upon the morphologies on the length scale. Liu *et al.* reported helical TiO_2_ nanofibres (with helix pitches over tens nm) which were constructed by the helical stacking of TiO_2_ NPs (∼20 nm) along the organic soft templates, thus creating a dissymmetric field to induce CD optical activity.^27^ Their DRCD and UV-Vis spectra for those helical TiO_2_/organic hybrids and anatase TiO_2_ products (obtained at 550 °C) were similar to our samples of PEI/tart@TiO_2_ and TiO_2_-500, respectively. Therefore, it can be said that the optical activity on TiO_2_ should be attributed to other reasons except the general helical morphologies. Indeed, chirality can be found in a broad size-scale from atomic/molecular-, to nano-/micro- and to macro-scale. It was even found that several kinds of chiral features (*e.g.*, lattice-chirality, shape-chirality) at different length-scales could simultaneously appear on the same chiral inorganic nanomaterial (*e.g.*, ZnO, HgS).^15,36^ In another example,^28^ twisted TiO_2_ nanoribbons were prepared by using organic assemblies as the templates. Both the as-obtained TiO_2_/organics hybrids and anatase TiO_2_ after calcinations showed the twisted morphologies on the nano-scale. When the twisted nanoribbons of anatase TiO_2_ were transformed into nanoparticles by grinding, CD optical activity remained still in the same signature. After heating at 700 °C, anatase TiO_2_ turned into rutile TiO_2_, accompanied with the transformation of twisted nanoribbons into nanofibres and nanoparticles. However, the rutile TiO_2_ was still optically active with accompanying the red shift both on the CD and UV-Vis absorption spectra from anatase to rutile (this is similar to our case shown in Fig. 5a). According to these results, they argued that chiral defects at the Angstrom level drive the optical activity of the anatase and rutile TiO_2_. In other chiral inorganic nanomaterials (*e.g.*, Ta_2_O_5_, CdSe/ZnS),^18,43^ some defects (*e.g.*, point defects, screw dislocations) were also considered to induce the chirality.

**Fig. 6:**
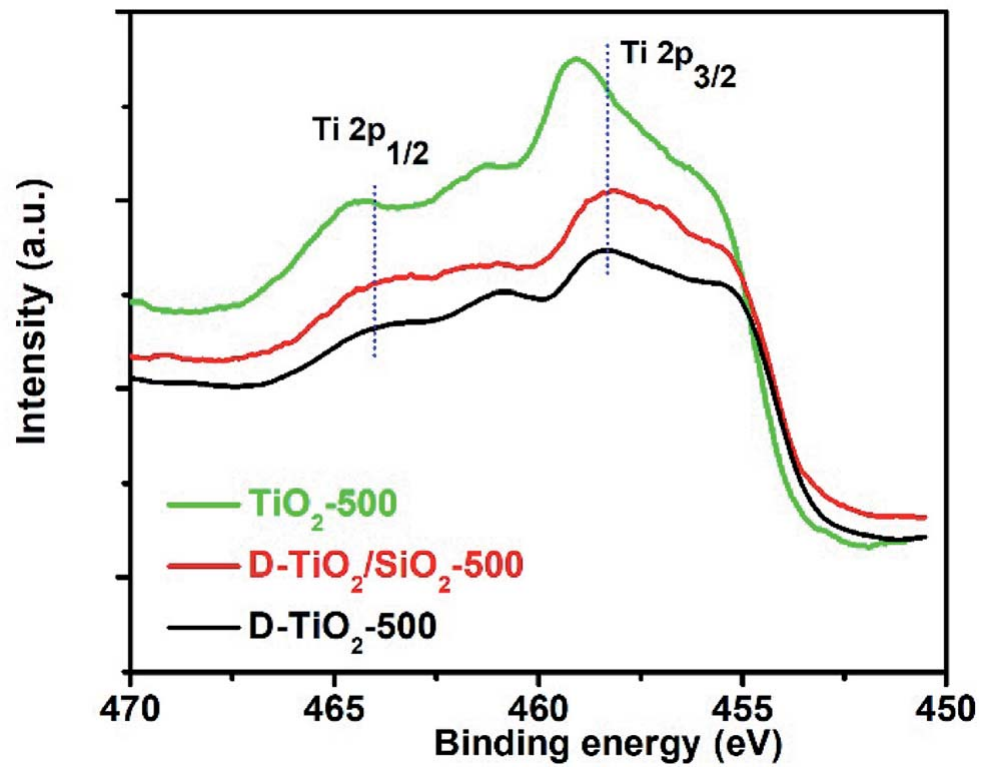
XPS spectra of the samples of d-TiO_2_ (black line), d-TiO_2_/SiO_2_ (red line), and achiral aTiO_2_ (green line) calcined at 500 °C.

In our very recent research on the chirality of chiral SiO_2_ nanofibres, it has been found that the sub-10 nm SiO_2_ NPs obtained by downsizing the long chiral SiO_2_ nanofibres *via* hydrothermal or chemical treatments were still chiral, as confirmed by antipodal signals corresponding to the Si–O–Si bond on the vibration circular dichroism spectra (VCD).^13,44^ Based on these results, we consider that the longer chiral SiO_2_ nanofibres would be constructed by linkage of a lot of small chiral (–O–Si–)_*n*_ clusters (<10 nm) which resemble molecular scale asymmetry with special conformation. Therefore, even without chiral outward shapes on the larger length-scale, the non-helical SiO_2_ nanofibres are chiral because they carried the chiral information on the clusters-like scale (<10 nm). It is of conclusive that similar to silica system, PEI/tart assemblies also effectively prompted the deposition of TiO_2_ to give chiral PEI/tart@TiO_2_ nanofibres. Even these nanofibres were broken into nanoparticles of rutile TiO_2_, CD optical activity was still preserved. Hence, it is suggested that the chiral transfer mechanism *via* hydrolytic condensation and the concept of chiral domains for PEI/tart@SiO_2_ could be similarly applied to PEI/tart@TiO_2_ because in the as-prepared state, both SiO_2_ and TiO_2_ is similarly amorphous. For the chirality origin of TiO_2_ and TiO_2_/SiO_2_-related samples, we think that the asymmetric arrangement of Ti and O atoms in small chiral domains (<10 nm) would be important. That is, the structural properties (*e.g.*, the lengths and angles of Ti–O bonds, coordination numbers and geometry of Ti–O units) of the initially formed Ti–O clusters around the surface of PEI/tart would be influenced by the chiral conformation of PEI/tart, which results in the formation of chiral domains (<10 nm) with asymmetric arrangement of Ti and O atoms in TiO_2_. To some extent, this speculation was supported by comparison of the Ti 2p XPS spectra of the samples of chiral d-TiO_2_, d-TiO_2_/SiO_2_ and achiral aTiO_2_ (prepared without tart, details see ESI‡) calcined at 500 °C (Fig. 6). Interestingly, the binding energy of the peak for Ti 2p_3/2_ (or Ti 2p_1/2_) for the chiral d-TiO_2_-500 is close to that of d-TiO_2_/SiO_2_-500 but was shifted toward higher region compared to the binding energy of the achiral aTiO_2_-500. Since the binding energy is sensitive to the local structural information (*e.g.*, the numbers and positions of oxygen atoms surrounding Ti atom, the length and angles of Ti–O bonds, the electron density around Ti cation), the shift on the binding energy between chiral TiO_2_ and achiral TiO_2_ implied that the structural difference of the coordination bonding of Ti–O between chiral TiO_2_ and achiral TiO_2_. The phase transformation was in fact a process of rearrangement of atoms, thus the local adjustment of Ti and O atoms in these chiral domains was unavoidable. Based on that the chiral TiO_2_ (or TiO_2_/SiO_2_) calcined at 500 °C showed the highest intensities of CD signals, it seems that these chiral structures of Ti–O clusters were thermodynamically metastable in a relatively low temperature range (about 500 °C). Therefore, even if there was a heating-driven structural self-adjustment of the initial chiral Ti–O clusters in the amorphous TiO_2_, the chiral feature of the as-formed Ti–O clusters in the anatase TiO_2_ were still maintained or even strengthened, as seen in the intensities of CD signals which increased from the samples before calcination to the ones calcined at 500 °C for both chiral TiO_2_ and TiO_2_/SiO_2_.

In principle, chiral Ti–O clusters should be different from the normal structures of Ti–O units in amorphous, anatase and rutile TiO_2_. In the 230 kinds of space groups of crystals, 65 kinds of Sohncke space groups can lead to chiral crystals.^45^ However, both the space groups of anatase (*I*4_1_/*amd*) and rutile (*P*4_2_/*mnm*) TiO_2_ contain some symmetric operations (*e.g.*, mirror, glide) and do not belong to the Sohncke space groups. Nevertheless, the small chiral Ti–O clusters initially formed in the amorphous TiO_2_ could work as chiral sources to produce new chiral Ti–O clusters (*i.e.*, chiral defects) in the crystalline anatase and rutile TiO_2_. In this way, the chiral information of Ti–O clusters was self-transferred *via* the structural maintenance or self-adjustment during the phase transformation of TiO_2_. Since these chiral domains were small, it was less influenced by the morphological change on the larger length-scale and thus optically active rutile and anatase TiO_2_ could be acquired at 800 °C. Nevertheless, it should be noted that more future work is necessary to reveal the detailed information of these chiral structures and the chirality origin.

## Conclusions

In summary, chiral TiO_2_ and TiO_2_/SiO_2_ could be easily prepared *via* hydrolytic condensation of water soluble titanium bislactate on chiral PEI/tart complexes which played as catalytic chiral templates. It was considered that the chirality of these chiral products is based on the asymmetric arrangement of Ti and O atoms in a small size scale less than 10 nm. Consequently, the chirality could be maintained even on rutile TiO_2_ calcined at 800 °C even the morphologies on the nano-/micro-scale were destructed. Because PEI/tart was also able to induce the deposition of chiral SiO_2_ on their surface, the as-formed SiO_2_ could disperse TiO_2_ NPs and then restrict the growth and phase transformation (from anatase to rutile) of TiO_2_ NPs when TiO_2_ and SiO_2_ were co-deposited on the PEI/tart. Thus chiral sub-10 nm anatase TiO_2_ NPs were well maintained and dispersed on the nanofibres at a high temperature up to 800 °C. In our preliminary test (unpublished), we have found that chiral TiO_2_ possesses better photocatalytic performance over achiral TiO_2_. Probably, the chirality on the atomic/molecular-scale may influence the properties of TiO_2_ and bring about new applications.

## Experimental

### Synthesis of PEI

The synthesis of PEI was performed in our previous work.^39^

### Synthesis of PEI/tart complex^40^

0.30 g of d-tartaric acid (d-tart) was added into 80 mL of H_2_O and heated around 80 °C. Meanwhile, 0.32 g of PEI powders were dissolved in 80 mL of H_2_O by heating around 80 °C with stirring. Then these two solutions were mixed with stirring for several minutes around 80 °C. After that, the mixed solution was placed into water bath and then cooled down to room temperature, and the pH of the solution was modulated to be ∼4.00 by diluted ammonia. The solution was placed in a refrigerator (∼4 °C) overnight to form a suspension, from which white precipitate was collected by centrifugation and further washed by H_2_O three times. The as-collected white products were the crystalline d-form PEI/tart complex (d-PEI/tart). For the l-form PEI/tart, the synthesis was the same to that of d-PEI/tart except replacing d-tart with l-tart.

### Synthesis of PEI/tart@TiO_2_ hybrids

The as-collected d-PEI/tart complex above was dispersed in 15 mL of H_2_O. The TiO_2_ source solution was prepared as follows: 6 mL of titanium bislactates (44 wt% aqueous solution, abbreviated as TiLact_2_, the commercial name is TC310 from Matsumoto Chemical Co. Japan), 6 mL of ammonia (1 mol L^−1^), and 8 mL of H_2_O was mixed with stirring for 30 minutes. Then the TiO_2_ source solution was added into the suspension of d-PEI/tart complex. After stirring for 2 hours at room temperature, white precipitates were collected by centrifugation, washed by H_2_O and acetone, and dried under vacuum. Finally, the white powders of d-PEI/tart@SiO_2_ were obtained. l-PEI/tart@TiO_2_ powders were similarly obtained by using l-PEI/tart complex.

### Synthesis of PEI/tart@TiO_2_/SiO_2_ hybrids

The d-PEI/tart complex above was dispersed in 15 mL H_2_O. The TiO_2_ source solution (6 mL of TiLact_2_ [44 wt% aqueous solution], 6 mL of 1 mol L^−1^ ammonia, 8 mL of distilled water) was prepared by the same way as shown above. The TiO_2_/SiO_2_ source solution was prepared by mixing the TiO_2_ source solution and 3 mL of tetramethoxysilane (TMOS) *via* stirring for 3 minutes and then added into the suspension of d-PEI/tart. After stirring of 2 hours, white precipitate was separated by centrifugation, washed by H_2_O and acetone, and finally dried under vacuum. The as-formed white powders were called as d-PEI/tart@TiO_2_/SiO_2_.

l-PEI/tart@ TiO_2_/SiO_2_ was similarly prepared by using l-PEI/tart complex.

### Synthesis of TiO_2_ and TiO_2_/SiO_2_ by calcination

PEI/tart@TiO_2_ and PEI/tart@TiO_2_/SiO_2_ were calcinated at a given temperature (500, 600, 700, 800 °C) for 1 h under air by which TiO_2_ and TiO_2_/SiO_2_ were formed, respectively. The products obtained at different temperatures was denoted as TiO_2_-*X* or TiO_2_/SiO_2_-*X* (*X* means the calcination temperature, *X* = 500, 600, 700, 800).

### Characterizations

XRD patterns were collected on a Rigaku RINT Ultima-III X-ray diffractometer with Cu Kα radiation (*λ* = 0.1540 nm). The SEM images were taken on a HITACHI SU8010 scanning electron microscope (SEM) equipped with energy dispersive spectrometer (EDS). The TEM analysis was finished on a HT7700 (Hitachi) instrument with acceleration voltage of 200 kV. The spectra of solid-state diffuse reflectance circular dichroism (DRCD) and UV-Vis absorption of the solid products (40 wt%) dispersed in KCl were simultaneously recorded on a JASCO J-820 spectropolarimeter equipped with a DRCD-466L unit. The TG analysis is conducted on a Exstar 6000 instrument (Seiko Instruments Inc.).

## Conflicts of interest

There is no conflicts to declare.

## Supplementary Material

RA-008-C8RA02926A-s001
