# Supplementary material for: Convenient chirality transfer from organics to titania: construction and optical properties
Source: RSC Adv. 2018 Apr 30;8(29):15951–60. doi: 10.1039/c8ra02926a (PMC9080238; doi:10.1039/c8ra02926a)
Supplement: RA-008-C8RA02926A-s001 [file RA-008-C8RA02926A-s001.pdf]

Electronic Supplementary Material (ESI) for RSC Advances.
This journal is © The Royal Society of Chemistry 2018

Supporting Information

Convenient Chirality Transfer from Organics to Titania: Construction and Optical Property

Xin-Ling Liu, Ken Murakami, Hiroyuki Matsukizono, Tsunega, Seiji and Ren-Hua Jin*

Department of Material and Life Chemistry, Kanagawa University,
3-2-7 Rokkakubashi, Yokohama 221-8686, Japan

*Corresponding author, E-mail: rhjin@kanagwa-u.ac.jp

Synthesis of achiral TiO_2

1. Synthesis of crystalline PEI template

0.474 g PEI powders were dissolved in 20 mL H_2O by heating around 80 ^oC with stirring. Then the hot PEI solution was placed at room temperature to form a suspension, from which white precipitate (i.e., crystalline PEI) was collected by centrifugation and further washing with H_2O three times. The as-obtained crystalline PEI above was dispersed in 15 mL H_2O.

2. Synthesis of achiral PEI@TiO_2 hybrids

The TiO_2 source solution was prepared as follows: 6 mL titanium bislactates (abbreviated as TiLact, the commercial name is TC310 from Matsumoto Chemical Co. Japan), 6 mL ammonia (1 mol/L), and 8 mL H_2O was mixed with stirring for 30 minutes. Then the PEI-containing suspension above was added into the TiO_2 source solution. After stirring for 2 hours at room temperature, white precipitate (i.e., achiral PEI@TiO_2) was collected by centrifugation, washing with H_2O and acetone, and drying under vacuum.

3. Synthesis of achiral aTiO_2 by calcination

The as-formed PEI@TiO_2 above was further calcinated at 500 ^oC (or 800 ^oC) for 1h under air, which led to achiral *a*TiO_2-500 (or TiO_2-800). The XRD patterns and SEM images of *a*TiO_2-500 and *a*TiO_2-800 are shown in Figure S1 and S2, respectively.

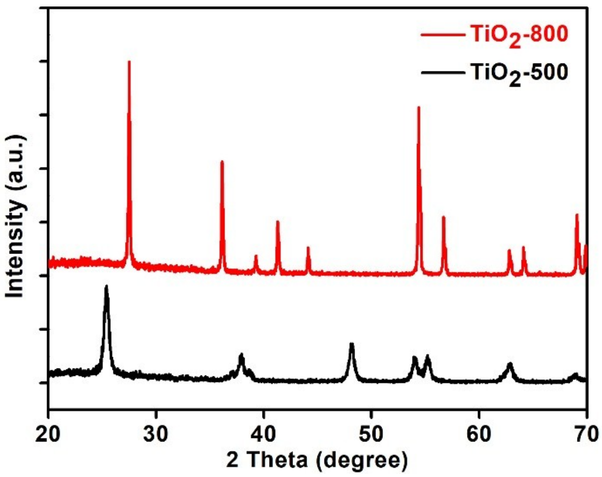

Figure S1. XRD pattern for the achial *a*TiO_2 after calcination at 500 ^oC (black line) and 800 ^oC (red line).

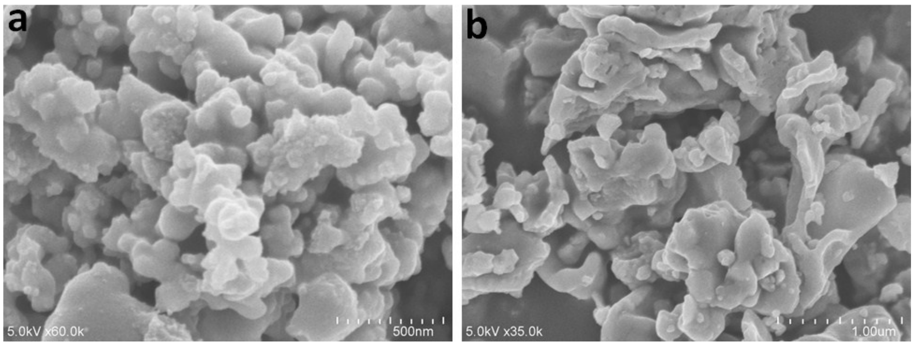

Figure S2. SEM images for the achial *a*TiO_2 after calcination at a) 500 ^oC and b) 800 ^oC.
